# Supplementary material for: Multifactorial motor behavior assessment for real-time evaluation of emerging therapeutics to treat neurologic impairments
Source: Sci Rep. 2019 Nov 11;9:16503. doi: 10.1038/s41598-019-52806-1 (PMC6848091; doi:10.1038/s41598-019-52806-1)
Supplement: Supplementary file 1 — Supplementary information [file 41598_2019_52806_MOESM1_ESM.pdf]

TITLE

Multifactorial motor behavior assessment for real-time evaluation of emerging therapeutics to treat neurologic impairments

AUTHORS

Riazul Islam, M.S.E., Carlos Cuellar, Ph.D., Ben Felmlee, Tori Riccelli, Jodi Silvernail, Suelen Lucio Boschen, Ph.D., Peter Grahn, Ph.D., Igor Lavrov, M.D., Ph.D.

SUPPLEMENTARY TABLES

Supplementary table 1: Components and cost of the BWS system**.**

Part	Quantity	Price ($)	Total ($)
Corner cubes	4	6.8	27.3
8020 Aluminum extrusion	2	31.5	63.1
Adjustable feet	4	13.3	53.4
Button head bolt	12	11.6	11.6
Steel rod	2	15.7	31.5
Aluminum Rod	2	25.6	51.3
Linear bearing	4	13.9	55.8
Stop collars	9	3.8	35.0
Rubber cushion	8	1.6	13.5
rod mount bolts	8	0.1	1.0
Spring	1	8.7	8.7
Miscellaneous	-	-	40
**Total price:**	-	-	**~ 400**

Supplementary table 2: Theoretical and experimental frictional force analysis of a single linear bearing

Theoretical calculation (N)		Experimental Measurement (N)
With Seals	Seals Removed	
2.003	0.0294	0.034±0.012(n=4)

Supplementary Table 3: Summary of computed kinematics and EMG parameters for PCA.

#	**Kinematic parameters**
1	Step height
2	Step length
3	Toe fluctuation
4	Step duration
5	Stance phase
6	Swing phase
7	Drag phase
8	Hip angle displacement
9	Knee angle displacement
10	Ankle angle displacement
11	MTP angle displacement
	**EMG burst parameters**
12	TA burst amplitude
13	MG burst amplitude
14	TA burst duration
15	MG burst duration
16	Co-activation period
	**Kinetic parameters**
17	F_x variation
18	F_y variation
19	F_z variation
20	T_x variation
21	T_y variation
22	T_z variation
	**Open field parameters**
23	Displacement x direction
24	Displacement y direction
	**fMEP parameters**
25	# of middle response (MR)- TA
26	# of late response (LR)-TA
27	MR area-TA
28	LR area-TA
29	MR area/LR area-TA
30	# of MR- MG
31	# of LR-MG
32	MR area-MG
33	LR area-MG
34	MR area/LR area-MG
